# 

**Published:** 1898-05

**Authors:** 


### CONTENTS.

PAGE

ORIGINAL ARTICLES:
The Relation of Veterinarians to the Public Health as Meat- and Milk-Inspectors. By DR. H. D. GILL . . . 295
Powdered Soap as a Cause of Death among Swill-fed Hogs. By VERANUS A. MOORE, B.S., M.D. . . . 306
The Municipal Control of Milk-supply in Cities and Villages. By W. H. HEATH, M.D. . . . 315

ABSTRACTS FROM FOREIGN JOURNALS:
The Effect of Tuberculin and of Glycerin-extract from Tuberculous Organs . . . 321
Discussion of Methods in Berlin for Fighting the Hoof- and Mouth-disease . . . 322
Macroscopically Visible Trichina . . . 322
Therapeutics of Wounds in the Joints and Ligaments . . . 322
The Sewing of Wounds . . . 323
Disinfection of Wounds and Instruments by a Solution of Formalin . . . 323
Prescriptions . . . 324

SELECTIONS:
Bandits who Make Society their Prey . . . 324

REPORTS OF CASES:
Purpura Hæmorrhagica in a Dog. By WILFRED LELLMAN, D.V.M. . . . 327
Clinical Reports—New York College of Veterinary Surgeons . . . 328
I. An Obscure Case of Lameness. II. Tetanus; Recovery. By W. G. HOLLINGSWORTH, D.V.S. . . . 329

AMONG THE PROFESSION IN NEW YORK STATE . . . 331
The Empire State Veterinary Colleges—New York Associations—What New York Veterinarians Want—What a New York House has Done . . . 336

CONTROL WORK . . . 343

EDITORIALS:
Our Empire State Edition . . . 344
Will New York Lead? . . . 344
A Centre of Veterinary Education . . . 344

MARRIAGES . . . 345
REVIEW . . . 346
PERSONALS . . . 346

SOCIETY PROCEEDINGS:
U. S. V. M. A.—District of Columbia—New York County—Iowa—Wisconsin—Missouri Valley . . . 351

COMMENCEMENT EXERCISES:
New York—Grand Rapids—United States—American . . . 359

LEGISLATION . . . 364
POINTS . . . 366

